# New Books

**Published:** 1865-12

**Authors:** 


New Books.—We are indebted to the publishers of medical books for a large number of most valuable works. We have only space to barely acknowledge the reception; we shall give fuller notice of them as early as possible; meanwhile our readers may be assured that the works noticed as "received," in the last number as well as the present, are all really valuable and almost indispensable books—books for the profession.

